# The Δ33-35 Mutant *α*-Domain Containing *β*-Domain-Like M_3_S_9_ Cluster Exhibits the Function of *α*-Domain with M_4_S_11_ Cluster in Human Growth Inhibitory Factor

**DOI:** 10.1155/2010/294169

**Published:** 2010-05-17

**Authors:** Qingui Bao, Zhichun Ding, Zhong-Xian Huang, Xiangshi Tan

**Affiliations:** ^1^Institute of Biomedical Science, Fudan University, 220 Handan Road, Shanghai 200433, China; ^2^Department of Chemistry, Fudan University, Shanghai 200433, China

## Abstract

Neuronal growth inhibitory factor (GIF), also known as metallothionein (metallothionein-3), impairs the survival and neurite formation of cultured neurons. It is known that the *α*-*β* domain-domain interaction of hGIF is crucial to the neuron growth inhibitory bioactivity although the exact mechanism is not clear. Herein, the *β*(MT3)-*β*(MT3) mutant and the hGIF-truncated Δ33-35 mutant were constructed, and their biochemical properties were characterized by pH titration, EDTA, and DTNB reactions. Their inhibitory activity toward neuron survival and neurite extension was also examined. We found that the Δ33-35 mutant *α*-domain containing *β*-domain-like M_3_S_9_ cluster exhibits the function of *α*-domain with M_4_S_11_ cluster in hGIF. These results showed that the stability and solvent accessibility of the metal-thiolate cluster in *β*-domain is very significant to the neuronal growth inhibitory activity of hGIF and also indicated that the particular primary structure of *α*-domain is pivotal to domain-domain interaction in hGIF.

## 1. Introduction

The pathological pattern of Alzheimer's disease (AD) is characterized by the progressive loss of neurons accompanied by the formation of intraneural neurofibrillary tangles and extracellular amyloid plaques [1]. One hypothesis postulated that these symptoms are caused by an imbalance of neurotrophic factors [2]. In 1988, Uchida and coworkers established that brain extracts from AD patients stimulate the neuron survival to a greater degree than normal brain extracts, due to the loss of a human growth inhibitory factor (hGIF) [3, 4]. It has also been reported that the metallothionein-3 (MT3) level decreases in astrocytes in lesioned areas of degenerative diseases such as Parkinson's disease, amyothrophic lateral sclerosis, and progressive supranuclear palsy [5]. Because of the high homogeneity in primary structure with MT1/MT2, hGIF was classified as a member of MT family and was also named hMT3 [6]. 

Metallothioneins (MTs) are low molecular weight, cysteinerich, and metal ion binding proteins. In mammalians, four MT isoforms (MT1, MT2, MT3, and MT4) have been identified [7]. The two major isoforms MT1 and MT2 are found in most organs and can be rapidly induced by a wide range of stimuli, such as metal ions and cytokines [8, 9]. However, MT3 and MT4 are specifically expressed in the central nervous system (CNS) and stratified squamous epithelia, respectively, and have no response to these inducers [7]. All of the mammalian MTs display two domains, and each domain contains a metal-thiolate cluster: the N-terminal *β* *-*domain contains a cluster of three metals coordinated with nine cysteines; the C-terminal *α*-domain contains a cluster of four metals coordinated with eleven cysteines. The cluster structure, where the metal ions are tetrahedrally coordinated by bridging and terminal cysteines, is important for the function of MTs [10]. Unlike most other metalloproteins, metallothionein can bind metals with a high thermodynamic stability [11–13]. These properties are believed to enable MTs also to function in essential trace metal homeostasis, detoxification of heavy metal ions and oxidative stress response [14, 15], and so forth. Despite the high similarities between the primary structures of MT-3 and mammalian MT-1/MT-2, the biological properties of these three proteins differ. The most conspicuous feature is that MT-3 but not MT-1/MT-2 exhibits a neuron growth inhibitory activity in neuronal cell culture studies. This activity of MT-3 has been reported for the native Cu_4_Zn_3_-hGIF and recombinant Zn_7_-hGIF [16]. Further studies have established that the inhibitory activity is mainly related to the N-terminal *β*-domain, whereas the C-terminal *α*-domain alone is found to be inactive [17, 18]. According to our previous studies, it is indicated that the neuronal growth inhibitory bioactivity of hMT3 is regulated by various factors. For examples, both the single amino acid insert (Thr) close to the N-terminus and a glutamate-rich hexapeptide insert close to the C-terminus are pivotal to the bioactivity of hGIF [6]. The CPCP motif is also key to the bioactivity; maybe this motif is the specific binding site of hMT3 with other biological molecules [17–19], the solvent accessibility of the metal-thiolate cluster, which is closely associated with the structure of the protein, and mutual accessibility of metal-thiolate clusters with biologically sensitive small molecules [20–23]. Moreover, domain-domain interactions maybe play important roles in modulating the stability of the metal-thiolate cluster and the conformation of the *β*-domain, and so forth. 

 The earlier research showed that *α*-domain is very significant to the biological function of the protein. We proposed that the *α*-domain has a stabilizing effect to *β*-domain in hGIF. However, the mechanism of this stabilizing effect remains unclear. In 2003, Dr. Wenhao Yu in our lab constructed C34M and C35S mutants of monkey metallothionein and discovered that the newly formed *α*-domain (residue 32 to 61) was able to combine with up to fourfold of metal ions, although the domain had only nine cysteines [24]. Besides, the number of cysteine in the *α*-domain of sheep metallothionein (sGIF), which was found in 2002, is nine, two less than other species which contain eleven cysteines. Hence, by comparing the primary structure of hGIF with sGIF, we constructed an hGIF Δ33-35 mutant (Δ33-35 mutant, the fragment of ^33^SCC^35^ in hGIF was removed, and it contains two M_3_S_9_ clusters as same as sGIF) and a *β*(MT3)-*β*(MT3) mutant. The biochemical properties of the mutants were characterized by pH titration, EDTA, and DTNB reaction, and their bioactivity toward neuron survival and neurite extension was also examined. We found that in hGIF Δ33-35 mutant the truncated *α*-domain (also named as *β*′-domain in this paper) contains M_3_S_9_ cluster which is similar to the wild type hGIF *β*-domain, while it exhibits the functions of *α*-domain with M_4_S_11_ cluster in human growth inhibitory factor. The experimental results showed that the stability and solvent accessibility of the metal-thiolate cluster in the *β*-domain is very significant to the neuronal growth inhibitory activity of hGIF and also indicated that the particular primary structure of the *α*-domain of hGIF is pivotal to domain-domain interaction in hGIF. 

## 2. Experimental

### 2.1. Reagents

 Fusion expression vector pGEX-4T-2, Escherichia coli strain BL21, glutathione Sepharose 4B, Superdex-75, and Sephadex G-25 were purchased from Amersham Pharmacia Biotech. The (deoxy-ribonucleoside triphosphate) dNTP, T4 DNA ligase, and restriction enzymes, *BamH I and EcoR I*, were purchased from New England Biolabs. Pfu DNA polymerase, cell culture reagents, isopropyl *β*-D-thiogalactoside (IPTG), and Triton-100 were purchased from Sangon (Shanghai, China). The DNA gel extraction kit was purchased from Qiagen. 2,2′-dithiodipyridine, 5,5′-dithiobis-(1-nitrobenzoic acid) (DTNB), ethylenediamine tetraacetic acid (EDTA), and thrombin were from Sigma (St. Louis, MO, USA). Neurobasal-A medium and B27 serum-free supplements were purchased from GIBCO-BRL (Gaithersburg, MD, USA). The other reagents were of analytic grade.

### 2.2. Cloning, Expression, and Purification of hMT3 and Its Variants

 Human MT3 (hMT3) cDNA was prepared from cells by reverse transcription followed by polymerase chain reaction (PCR). The genes of the *β*(MT3)-*β*(MT3) mutant, the hGIF Δ33-35 mutant, and the single *β*-domain of hMT3 were amplified by PCR method [25]. Each segment was digested and cloned into vector pGEX-4T-2 as a *BamH I/EcoR I *fragment and verified by DNA sequencing. The expression and purification procedure for hMT3 and its mutants were carried out as described in the instructions for Glutathione-Sepharose 4B (GE healthcare life science) with some modifications. Briefly, cultures of cells (50 mL) were grown overnight in LB medium containing 100 mg mL^−1^ of ampicillin at 37°C. This start culture was diluted 100-fold into 500 mL of fresh 2*·*YTA, and the cells were induced with 0.1 mM IPTG when an absorbance of 0.6–0.8 at 600 nm was attained, followed by 1-hour growth before addition of 0.2 mM ZnSO_4_. The cells were harvested at OD_600_ value of 2.5, which was generally reached 3-4 hours after induction. Then, under protection of mercaptoethanol, the GST fusion protein was separated by affinity column of Glutathione Sepharose 4B, which was washed by PBS until the absorbance at 280 nm was less than 0.02. Then the fusion protein was digested by thrombin for 14 hours at 25°C in a cleavage buffer (50 mM Tris-HCl, 150 mM NaCl, and 2.5 mM CaCl_2_, pH 8.0). The elute containing thrombin and recombinant hMT3 (tagged Gly-Ser in the N-terminus) cleaved from the fusion protein was pooled, concentrated, and further separated by a Superdex-75 gel filtration column equilibrated with 10 mM Tris-HCl, 50 mM NaCl, pH 8.0. The main eluted peak was concentrated, desalted, lyophilized, and stored at −80°C.

### 2.3. Protein Characterization and Reconstitution

The apo-form of hMT3 and its variants were generated according to literature [26] and fully Cd^2+^- or Zn^2+^-loaded proteins were prepared by reconstitution under the protection of nitrogen [26]. The concentration of MTs was assessed by reacting with 2, 2′-dithiodipyridine in 10 g L^−1^ SDS, 1 mM EDTA, and 12 mL L^−1^ acetate (pH 4.0), using *ε*_324_ = 19,800 M^−1^cm^−1^ [27]. The content of metal ions in each protein was analyzed by flame atomic absorption spectrophotometry (WFX-110, BRAIC, Beijing, China). Electrospray ionization mass spectrometry (ESI-MS) was used to measure the molecular weights of hMT3 and its variants. Each protein was dissolved in 1% formic acid (v/v) at a concentration of 10 ng mL^−1^. The measurement was carried out on a Bruker Esquire 3000 electrospray mass spectrometer (Bruker Daltonics, Bremen, Germany). The instrumental conditions were capillary volt, 4 kV; dry gas, 5 L min ^−1^; nebulizer gas, 15 PSI; and infusion flow rate, 3 *μ*L min ^−1^.

### 2.4. UV-Vis and Circular Dichroid Spectra

 The UV-vis absorption spectra were scanned from 200 to 400 nm on a HP8453 UV-vis spectrophotometer (Hewlett-Packard, Palo Alto, CA, USA) at room temperature using a 1.0 cm quartz cuvette. CD spectra were measured in the range of 200–300 nm on a Jasco J-715 spectropolarimeter at room temperature in phosphate buffer (10 mM Tris-HCl, 100 mM KCl, pH 8.0).

### 2.5. pH Titration

 The spectrophotometric pH titration was performed according to the method of Winge and Miklossy [10]. Briefly, 6-7 *μ*M Cd^2+^-reconstituted proteins were dissolved in 10 mM Tris-HCl, pH 8.0, containing 100 mM KCl and titrated with increasing amounts of 1 M HCl. The progress of acidification was monitored at 250 nm on the HP8453 UV spectrophotometer.

### 2.6. Reaction with EDTA and DTNB

 The reactions of Cd^2+^-reconstituted hMT3 and its mutants with EDTA were investigated at 25°C according to the method of Li et al. [28]: 9 *μ*M protein was reacted with 1.2 mM EDTA in 10 mM Tris-HCl, pH 8.0, containing 100 mM KCl. To avoid the absorbance of EDTA, the reaction was monitored at 265 nm on the HP8453 UV spectrophotometer at 20-seocnd intervals for 100 minutes. The reactions of Cd^2+^-reconstituted hMT3 and its mutants with DTNB were studied according to the method of Shaw et al. [29]: 3.5 *μ*M protein was reacted with 1 mM DTNB in 10 mM Tris-HCl, pH 8.0, containing 100 mM KCl at 25°C. The reaction was monitored at 412 nm (*ε*_412_ = 13,600 mol^−1^cm^−1^) on an HP8453 UV spectrophotometer at 20-second intervals for 60 minutes.

### 2.7. Preparation of Rat Brain Extract

 Rat brain extracts were prepared as described previously [3]. Briefly, the whole brain from adult male Hooded Wistar rat (~200 g) was removed and homogenized in one volume of Hanks buffer, followed by centrifugation at 100× g. The supernatant was filter-sterilized (0.22 *μ*m filter, Millipore, Billerica, MA, USA). The total protein concentration of freshly prepared rat brain extract was determined by the Bradford method, and brain extracts were used immediately at a concentration of 150 *μ*g mL^−1^ [30].

### 2.8. Culture of Cerebral Cortical Cells

 Cultures of cerebral cortical cells were prepared as described previously [30, 31] with slight modification. Briefly, cerebral cortices of Wistar rat fetuses (day 18) were removed and cells were dissociated mechanically by passing coarsely minced cortical tissues gently and repeatedly through a pipette. Tissue debris was removed by gently passing through a filter. Cells were washed and suspended in a specific culture medium developed for selective neuronal growth, consisting of Neurobasal-A medium (GIBCO), 0.1% (f/c) B-27 supplement (GIBCO), and 0.1 mM (f/c) L-glutamine (Sigma). Then the cells were seeded on polylysine-coated 24-well culture plates at a density of 1 × 10^5^ cells/well. Cultures were maintained in a 37°C chamber under an atmosphere of humidified air containing 5% CO_2_. Four hours later, the culture medium was replaced with fresh medium containing with 150 *μ*g mL^−1^ rat brain extract and 10–20 *μ*g mL^−1^ proteins. Cultures were maintained under the same conditions for three days. To visualize neuronal cells, cultures were fixed in 4% paraformaldehyde and then stained with trypan blue. Finally, the neuron neurite length was determined by measuring the distance between the end of the neurite and the cell surface using the Leica Qwin program (Rueil Malmaison, France) and at least 60 neurons with more than 100 neurites were recorded in each individual experiment.

## 3. Results and Discussion

**Figure 1:**
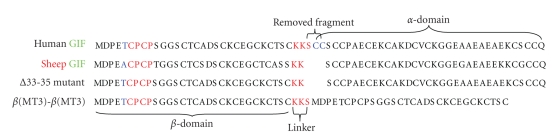
Amino acid sequence alignments of sheep MT3, human MT3, and its mutants.

DNA sequencing showed that all mutation genes were successfully constructed, and the primary structure of hMT3 and its mutants were shown in Figure 1. After expression and purification, the yields of recombinant proteins were about 9 mg L^−1^ culture. Since these proteins were cleaved from GST fusion proteins by thrombin, they had an additional Gly-Ser dipeptide in the N-terminus. However, the existence of the additional dipeptide does not obviously affect the structure and properties of the protein [31]. Their molecular weights were confirmed by ESI-MS, and the measured molecular weights were in good match with the theoretical values (Table 1). According to previous studies, Cd^2+^ is considered to be a useful probe of Zn^2+^ binding sites in MTs [32, 33], providing a wealth of structural information on the Zn^2+^-substituted MTs. Furthermore, the Cd^2+^-substituted MTs show a number of advantages over the Zn^2+^-substituted MTs, including a tendency toward higher oxidation stability, pronounced and thus easier to detect and quantify LMCT band, as well as the use of the ^111^Cd or ^113^Cd isotopes for NMR experiments, and so forth. Thus, Cd^2+^ has frequently been used as a substitute for Zn^2+^ in structural studies of hMT3 [20, 23, 34, 35]. In the present studies, Cd^2+^-reconstituted MTs were adopted in both spectroscopic and metal-binding studies, while Zn^2+^-reconstituted MTs were only used in bioassays, because Cd^2+^-MT is cytotoxic to the neurons in the bioassays [17]. The metal contents of hMT3 and its variants were determined by flame atomic absorption spectrophotometry and are listed in Table 1. 

**Figure 2:**
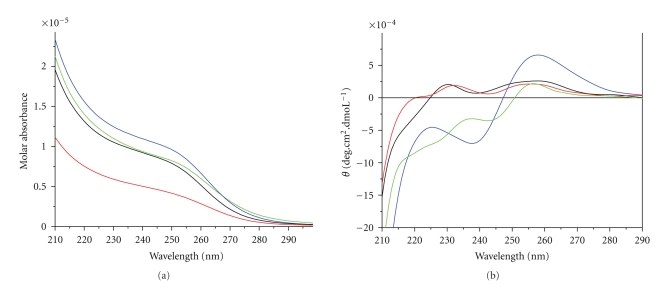
The UV (a) and CD (b) spectra of Cd^2+^-reconstituted hGIF (*blue line*), the Δ33-35 mutant (green line), the *β*(MT3)-*β*(MT3) mutant (black line), and the single *β*-domain of hGIF (red line). All proteins are dissolved in 10 mM Tris-HCl, 100 mM KCl (pH 8.0).

Figure 2(a) shows the UV-vis spectra of Cd^2+^-reconstituted hMT3 and its mutants, dissolved in 10 mM Tris-HCl, pH 8.0, containing 100 mM KCl. All the spectra showed an absorption shoulder at about 250 nm, which is the character of ligand-to-metal charge transfer (LMCT) of Cd-S bond [36]. The absorption at 280 nm was pretty low because of the absence of aromatic amino acids. According to the experiment result, the absorption intensity at 250 nm is in good line with the amount of Cd-S bond, indicating that the measurement of metal contents is accurate. 

CD spectrum is a common way to investigate the metal center structure and protein secondary structure. In the study of Metallothionein, CD is applied in the research of the geometry and polypeptide folding secondary structure. Figure 2(b) shows the CD spectra of hGIF, the *∆*33-35 mutant, the *β*(MT3)-*β*(MT3) mutant, and the single *β*-domain protein. The CD spectrum of hGIF agrees well with reporters [20]. It is known that the two absorption peaks ((260 nm (+) and 240 nm (−)) located at the low-energy region of Cd_7_-MT CD are signs of the formation of metal-thiolate cluster, and the two peaks are very sensitive to the variations of the structure of metal-thiolate cluster [13]. Compared to hGIF, the two peaks of the *β*(MT3)-*β*(MT3) mutant diminished dramatically, and the wavelength is blue shifted, making the shape of the *β*(MT3)-*β*(MT3) mutant similar to that of the single *β*-domain. It is reasonable by considering the previous research result that *α*-domain contributes mainly to the CD spectrum of Cd_7_-MT while *β*-domain has limited contribution. This is mainly because the three metal atoms of the *β*-domain locate in the same plane; therefore they are with high symmetry and contribute to a small CD absorption. The N-terminal and C-terminal of the *β*(MT3)-*β*(MT3) mutant both formed a three-metal cluster and resulted in the weaker intensity of CD spectrum absorption than that of hGIF. As for the *∆*33-35 mutant, the two peaks located in low-energy region blue shifted evidently compared to hGIF. Besides, the Molar ellipticity of CD spectrum of the *∆*33-35 mutant decreases significantly, and the shape of CD spectrum is closer to single *β*-domain. Thus, it is very likely to form two three-metal clusters, M_3_S_9_, in the *∆*33-35 mutant [35]. However, there is clear distinction in the shapes of CD spectra between the *∆*33-35 mutant and the single *β*-domain, which demonstrates some discrepancy existed in the *β*′-domain of the *∆*33-35 mutant and the *β*-domain of hGIF. Maybe the different distribution of the cysteine in the peptide chain leads to the change of protein folding patterns and the structure of metal-thiolate cluster, eventually causing the change of CD spectrum.

**Figure 3:**
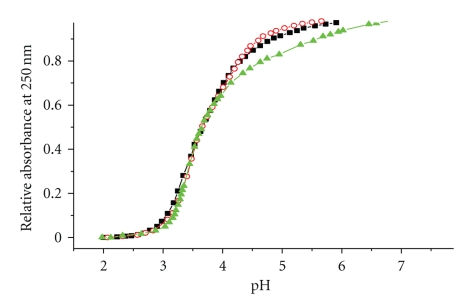
UV absorbance at 250 nm of pH titration of Cd^2+^-reconstituted hGIF (*solid squares*), the *∆*33-35 mutant (*open circles*), and the *β*(MT3)-*β*(MT3) mutant (*solid triangles*). All proteins are dissolved in 10 mM Tris-HCl, 100 mM KCl, and pH 8.0.

In the experiment of pH titration, the concentration of protons would increase with the decrease of pH. So, protons would compete with thiolate ligand for binding to Cd^2+^, which leads to the release of Cd^2+^from the Cd^2+^-thiolate cluster, causing the declining of the characteristic absorption at 250 nm. Hence, the UV absorbance at 250 nm was recorded during the pH titration in order to investigate the stability of the Cd-S clusters. According to our previous study, the titration curve of hMT3 was not clearly divided into two independent stages as it is difficult to tell one stage from the other in the pH titration plot. It is indicated that there is no sharp stability difference between the two Cd-S clusters. According to Figure 3, there is an obvious distinction between the pH titration curves of the *β*(MT3)-*β*(MT3) mutant and hGIF. Due to lacking in *α*-domain in the *β*(MT3)-*β*(MT3) mutant, the stability of Cd_3_S_9_ in the *β*(MT3)-*β*(MT3) mutant begins to decrease from neutral pH, while the stability of Cd_3_S_9_ begins to decrease from pH 5 in *∆*33-35 mutant and hGIF. This further proves that *α*-domain can stabilize the *β*-domain effectively in hGIF. Compared the pH titration curves of the *∆*33-35 mutant to the curves of hGIF, we found that the deletion of two cysteines in *α*-domain did not influence the stability of the Cd_3_S_9_ cluster to any obvious degree in hGIF. As we all know, both the *∆*33-35 mutant *β*′-domain and the *β*(MT3)-*β*(MT3) mutant *β*-domain contain a Cd_3_S_9_ cluster, while there is a Cd_4_S_11_ cluster in the hGIF *α*-domain. Interestingly, the stabilizing function of Cd_3_S_9_ in *β*′-domain is more similar to the Cd_4_S_11_ cluster in hGIF *α*-domain. This result becomes reasonable when the primary structures of the *β*′-domain and the hGIF *α*-domain are considered since they have the same primary structure except for the removed fragment (^33^SCC^35^). Thus, we suggest that probably it is due to the particular primary structure of the *α*-domain they contain, which stabilized the *β*-domain through the hydrogen bond or hydrophobic interaction, that play an important role in the domain-domain interaction as we described in the previous study [21].

The reaction of MTs with EDTA reflects competition between the sulfhydryl group and the exogenous ligand in the binding of metal ions and is also used to investigate the stabilities of metal-thiolate cluster [35]. Under pseudofirst-order conditions (concentration of EDTA is 300 times that of the MTs), this reaction for hMT3 and its mutants is obviously biphasic, with a fast phase and a slow phase. The observed rate constants were obtained by plotting ln (*A*_*t*_ − *A*_∞_) versus time and are listed in Table 2. As shown in the table, the velocity of *β*(MT3)-*β*(MT3) two-phase reaction increased obviously, while that of the *∆*33-35 mutant and hGIF is similar in magnitude, which means that the stability of the metal-thiolate cluster of the *∆*33-35 mutant has no considerable change compared to that of hGIF. This result is consistent with the pH titration experiment result. Unlike EDTA, DTNB can react with the nucleophilic sulfhydryl groups in MTs. This reaction is closely related to the solvent accessibility of the metal-thiolate cluster and usually is biphasic for MTs [29]. According to Winge and coworkers, the fast and slow phases may correspond to the reaction of DTNB with the *β*-domain and the *α*-domain of MTs, respectively [17]. The observed rate constants were obtained by plotting ln (*A*_∞_ − *A*_*t*_) versus time and these are also listed in Table 2. As indicated, the fast reaction rate constant of the *β*(MT3)-*β*(MT3) mutant was almost twice that of hMT3, showing that the solvent accessibility of the Cd_3_S_9_ cluster in the *β*-domain of the *β*(MT3)-*β*(MT3) mutant was greatly enhanced. However, the fast reaction velocity of the *∆*33-35 mutant with DTNB is closer to that of hGIF, as well as the slow reaction velocity. This means that the solvent accessibility of the two Cd_3_S_9_ clusters in the *∆*33-35 mutant is similar to its counterpart in hGIF. In regards with the results of EDTA and DTNB reactions, the Cd_3_S_9_ cluster in the *β*′-domain of the *∆*33-35 mutant, has the same stability and solvent accessibility as the Cd_4_S_11_ cluster in the hGIF *α*-domain. That means the structure of metal-thiolate cluster in hGIF *α*-domain will not change dramatically after removing two cysteines. Besides, compared to hGIF, the metal-thiolate cluster in *β*(MT3)-*β*(MT3) mutant shows lower stability and higher solvent accessibility. The most conspicuous difference between the *β*(MT3)-*β*(MT3) mutant and the *∆*33-35 mutant is that there is no particular amino acids sequence in the *β*(MT3)-*β*(MT3) mutant as that in hGIF *α*-domain. Thus, it once again proved that the primary structure of its hGIF *α*-domain is important to the function, which is quite in consistence with our pH titration experiment. 

**Figure 4:**
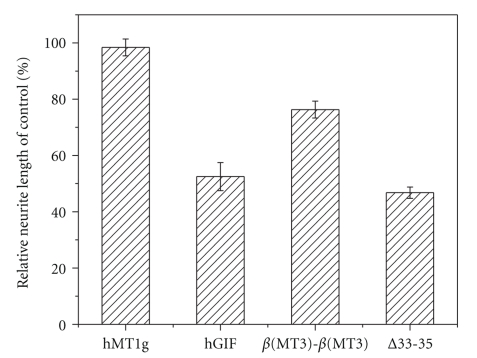
Effect of Zn^2+^-reconstituted hGIF, the *∆*33-35 mutant, and the *β*(MT3)-*β*(MT3) mutant on neurite extension in the presence of adult rat brain extract (150 *μ*g/mL) after 3 days, determined by the average neurite length of 60 neurons. As a comparison, the result of hMT1g (a human MT1 isoform where the segment is ^20^KCK^22^) under the same conditions was also shown. Error bars represent standard error values.

As mentioned above, Cd^2+^ recombinant protein is toxic to nerve cells. Thus, Zn^2+^-recombinant protein is adopted to test the physiological function. The nerve grow inhibiting function of hMT3, the *β*(MT3)-*β*(MT3) mutant, and *∆*33-35 mutant was tested. As shown in Figure 4, the average total length of hMT1g-treated neurites at three days was 203 ± 8 *μ*m. hMT3 significantly reduced the average neurite length to 94 ± 4 *μ*m. The average neurite lengths of *β*(MT3)-*β*(MT3) and *∆*33-35 mutant-treated neurons were 150 ± 5 and 92 ± 3 *μ*m, respectively (the ± value represents the standard error value). The neuronal growth inhibitory activity of hMT1g and hMT3 well agrees with that reported previously [31, 35]. Moreover, the *β*(MT3)-*β*(MT3) mutant has apparent lower inhibiting activity than that of hGIF. It would be much lower, if taking the concentration of *β*-domain into account that, the *β*(MT3)-*β*(MT3) mutant protein is two times more active than that of equal molar hMT3 in the nerve growth inhibition. However, the *∆*33-35 mutant is similar to the hGIF in neuronal growth inhibitory activity.

 It has been reported that the neuronal growth inhibitory activity of hMT3 mainly arises from its *β*-domain, while the *α*-domain is not directly involved in neuronal growth inhibitory activity [17]. However, the *α*-domain can affect the function of *β*-domain though domain-domain interactions do exist in MTs [21, 37–39], and it has been proved in our previous work [21, 37]. Taking all the experimental results together, for hGIF and its mutants, the stability and solvent accessibility of the metal-thiolate cluster in N-terminal *β*-domain are in good line with the bioactivity of the whole protein; so they may be very important to the neuronal growth inhibitory activity of hMT3. Probably, *α*-domain can influence the stability and solvent accessibility of the metal-thiolate cluster in *β*-domain through domain-domain interaction, eventfully effecting the bioactivity of the whole protein.

## 4. Conclusion

Considering the experimental result of EDTA, DTNB reaction, and pH titration together with the neuronal growth inhibitory activity, we proposed that the stability and solvent accessibility of the metal-thiolate cluster in *β*-domain is very significant to the neuronal growth inhibitory activity in hGIF. Comparing the *β*-domain of the *β*(MT3)-*β*(MT3) mutant and the *β*-domain of the *∆*33-35 mutant, these two mutants have different biological activities. It is precisely because the *∆*33-35 mutant has the *β*′-domain which is similar to hGIF *α*-domain. This further demonstrates that *α*-domain stabilizes *β*-domain via the domain-domain interaction in hGIF, thus to regulate the physiological activity. Moreover, in the present study, we found that there was an obvious difference between the *∆*33-35 mutant and the *β*(MT3)-*β*(MT3) in spectroscopy and biochemical properties. The *β*′-domain of the *∆*33-35 mutant, which contains M_3_S_9_ cluster, has the same metal-thiolate cluster as the *β*-domain of *β*(MT3)-*β*(MT3) mutant and still reserves the function of the M_4_S_11_ cluster of the hGIF *α*-domain. This indicated that the removing of two conservative cysteines does not lead to great changes of the metal-thiolate cluster in the hGIF *α*-domain. Meanwhile, the *β*′-domain of the *∆*33-35 mutant is obviously different from the *β*-domain of the *β*(MT3)-*β*(MT3) mutant in the stabilizing effect, of which the most conspicuous difference is that the *β*′-domain of the *∆*33-35 mutant contains the primary structure of the hGIF *α*-domain. But the *β*-domain of *β*(MT3)-*β*(MT3) mutant contains the primary structure of the hGIF *β*-domain. That indicated that the particular primary structure of *α*-domain is pivotal to domain-domain interaction in hGIF. We believe that our results will provide a step further toward better understanding of the relationship between the structure of metal-thiolate cluster and the bioactivity of this group of fascinating molecules.

## Figures and Tables

**Table 1:** Mass spectrometry analysis and metal contents of hGIF and its mutants.

Protein	*M* *w* (calc.)^a^	*M* *w* (meas.)^b^	Metal content
hGIF	7,071	7,070.7	7 ± 0.1
Δ33-35 mutant	6,778	6,778.6	6 ± 0.3
*β*(MT3)- *β*(MT3)	6,741	6,743.21	6 ± 0.2
Single *β*-domain	3,408	3,407.18	3 ± 0.2

^a^ The theoretical molecular weight.

^b^ The measured molecular weight.

**Table 2:** Observed rate constants of reaction of MTs with EDTA and DTNB.

Protein	Observed rate constants of reaction of MTs with EDTA		Observed rate constants of reaction of MTs with DTNB	
	*k*_*f*_ (×10^−4^ s^−1^)	*k*_*s*_ (×10^−5^ s^−1^)	*k*_*f*_ (×10^−3^ s^−1^)	*k*_*s*_ (×10^−4^ s^−1^)
hGIF	12.1 ± 0.3	4.2 ± 0.2	2.4 ± 0.1	9.1 ± 0.2
*∆*33-35	12.5 ± 0.2	3.8 ± 0.2	2.5 ± 0.1	8.8 ± 0.2
*β*(MT3)-*β*(MT3)	28.7 ± 0.3	5.8 ± 0.3	4.4 ± 0.2	15.6 ± 0.2
